# Anchoring Biodiversity Information: From Sherborn to the 21^st^ century and beyond

**DOI:** 10.3897/zookeys.550.7460

**Published:** 2016-01-07

**Authors:** Ellinor Michel

**Affiliations:** 1 Department of Life Sciences, The Natural History Museum, Cromwell Road London SW7 5BD, UK

Charles Davies Sherborn provided the bibliographic foundation for current zoological nomenclature with his magnum opus *Index Animalium*. In the 43 years he spent working on this extraordinary resource, he anchored our understanding of animal diversity through the published scientific record. No work has equaled it and it is still in current, and critical, use.

**Figure 1.**
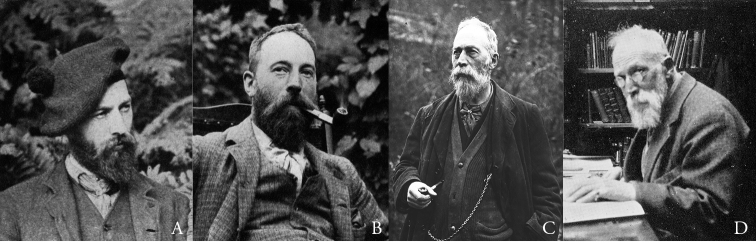
Charles Davies Sherborn aged 25 (**A**), 32 (**B**), 61 (**C**) and 72 (**D**).

Until now, Sherborn’s contribution has been recognized and relied upon by professional taxonomists worldwide but he has escaped the celebration of his accomplishment that is his due. This changed on 28 October 2011, with a symposium held in his honour at the Natural History Museum (NHM), London, on the 150^th^ year of his birth. The symposium was organized by the International Commission on Zoological Nomenclature (ICZN), in collaboration with the Society for the History of Natural History (SHNH). The full-day meeting included an international panel of experts on bibliography and biodiversity bioinformatics who linked a view of the past with an active debate on the future of these related fields. There were fifteen talks from distinguished speakers from around the world, and ten posters, including an exhibition of ‘Sherborniana’, or artifacts from Sherborn’s tenure at the NHM. This volume expands on that meeting, with contributions from most of the presenters and selected additional contributors. The global and temporal reach of this event was extended through high quality recordings of all the talks, posters and discussion, including slides and poster downloads, through this site: http://backdoorbroadcasting.net/2011/10/anchoring-biodiversity-information-from-sherborn-to-the-21st-century-and-beyond/ and videos of all the talks through http://www.iczn.org/Sherborn.

The papers in this volume fall into three general areas. In the first section, seven papers present different facets of Sherborn as a man, scientist and bibliographer, and describe the historical context for taxonomic indexing from the 19^th^ century to today. In the second section, five papers (with a major appendix) discuss current tools and innovations for bringing legacy information into the modern age. The final section, with three papers, tackles the future of biological nomenclature, including innovative publishing models and the changing tools and sociology needed for communicating taxonomy.

Because this volume is being produced as both a bound book and set of independent, Open Access papers free to download from the Web, there is a degree of overlap in some of the material covered. The papers need to be able to stand on their own, as well as to weave in to the whole overview of the accomplishments of this great man, his legacy and the roadmap for the future. In addition, because of the varied topics, the papers vary in style and length, some being more literary, some historical, some technical and some philosophical. Some are richly illustrated, others not at all. The only instructions to the authors were to attempt to reference each other’s papers to the greatest practical degree, simulating the kind of cross-topic communication one might have by being present at a symposium. The papers were all peer reviewed - most had critical input from three independent specialists in the field. I hope this diversity of approaches, rigorous oversight and the cross-pollination make the volume stimulating to read as a whole.

## Sherborn as a person, scientist and bibliographer, and his context

What kind of person takes on such a herculean task as did Sherborn? What was the source of his motivation? What were the related predecessors and descendants of his work? The first three papers address the whole of Sherborn’s life, and historical context, with a focus on *Index Animalium*. While Sherborn’s fame is based on *Index Animalium*, he undertook a number of other ambitious projects. Several of these also had lasting impacts on their respective fields. These are addressed in the next four papers in this section.

In ‘Charles Davies Sherborn and the “Indexer’s Club”’ Neal Evenhuis provides personal and highly sympathetic insights into the incredible drive and bibliographic skills Sherborn had to harness in his effort to make an essentially universal index to all animal names. Evenhuis served as a Commissioner and President of the ICZN for many years, and is a self-described ‘index-aholic’ – he knows whereof he speaks in understanding Sherborn’s motivation. As a plenary talk, and in this volume’s cornerstone paper, Evenhuis sets the tone. His wry wit makes Sherborn’s labours seem a natural endeavour, at least for those of an ambitious and altruistic mindset, motivated by the greatest of tasks, getting control over our information on the living world. Evenhuis also outlines forerunner and descendent projects of a similar nature.

Karolyn Shindler provides another, more personal portrait of Sherborn, adding a richness of experience of additional major projects in his life. Key among these was Sherborn’s involvement with the archives of Sir Richard Owen, the great anatomist and founder of the British Museum (Natural History), now the Natural History Museum, London. She highlights the key phrase that Sherborn used for himself, but perhaps applies to most collections and bibliophilic workers: ‘A Magpie with a Card Index Mind’. Although the historical facts that frame all the essays on Sherborn are the same, I found that the feeling for the man was quite different in each one. Shindler’s piece brought me practically to tears with awe and appreciation for Sherborn’s challenges and determination, but also feeling for his quirks. After reading it, I felt I had met the man himself.

Gordon McOuat’s contribution provides a overview of the evolution of nomenclatural codes and controversies in the decades around Sherborn, bringing the history of science to life. His contribution has a number of key messages on the relationships between names (dubbing) and meanings (taxonomy), on the struggle between establishing nomenclature tied to rules (codes) or to specimens (the type concept and museum catalogues). These issues were intensely addressed in the early and mid-19^th^ century and Sherborn’s magnum opus played a foundational role in establishing the systems we now use for all biology, not just zoology. Nonetheless many taxonomists today continue to befuddle these relationships, often through lack of knowledge of the long history of the discussions.

C. Giles Miller delves in to the scientific starting point for Sherborn’s indexing focus. Appropriately, this grew from taxonomic and collections work in the Natural History Museum on fossils, one of Sherborn’s early research loves. However, Miller provides a telling comment, saying that while Sherborn’s foraminiferan collections were respectable, they were not groundbreaking. In contrast, his bibliographic and indexing contributions changed the practice of micropalaeontology in the Natural History Museum, and thus the world. Moreover, his experience with this micropalaeo work set him on his future trajectory of ambitious indexing for all animal names and museum collections. It provided the focus for Sherborn to see his life’s calling.

**Figure 2.**
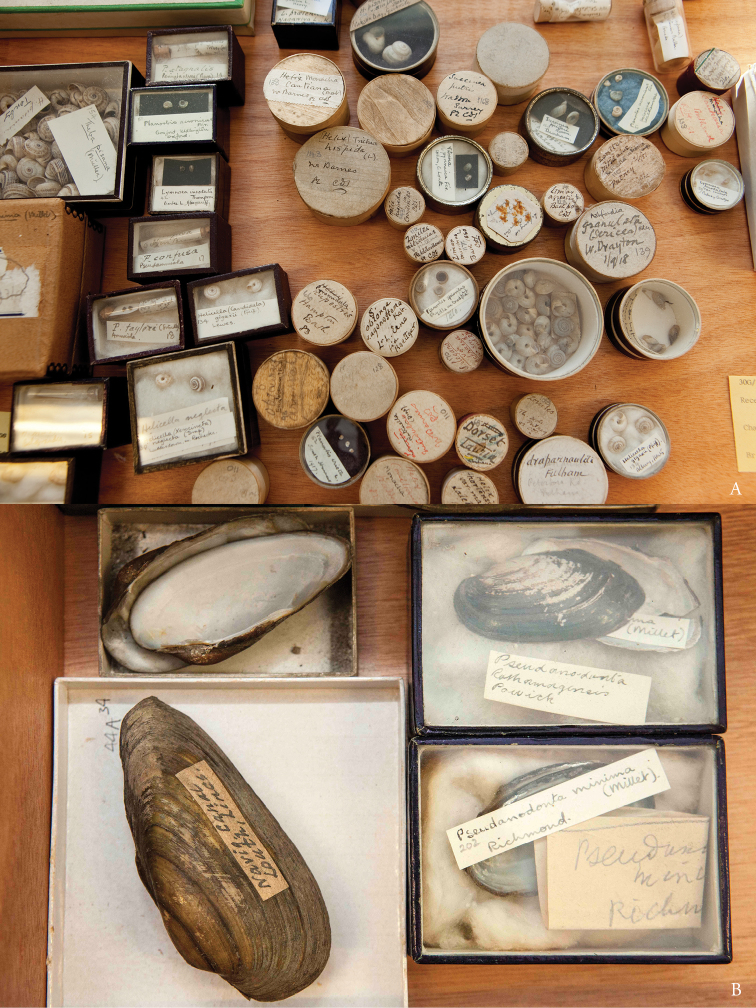
Sherborn’s land and freshwater mollusc collections, with specimen labels in his own handwriting **A** Gastropods **B** Bivalves.

As an aside, it should be noted that Sherborn had several additional scientific and collections interests not dealt with in the papers in this volume. Notably, his background in malacology resulted in three drawers of collections made by him, held at the NHM, London (Fig. 2). There are also collections of coins and stamps, apparently held in the collections of the British Museum.

The final major work undertaken by Sherborn at the end of his life was a listing of natural history collections that was actually titled ‘*Where is the damned collection*?’. Clearly, Sherborn had a sense of humour! Michael A. Taylor gives a vibrant description of this work – how it came about, the business hurdles and social controversies around getting it published, and the rewards for subsequent museum collections work. It makes surprisingly compelling reading to learn about Sherborn’s uncompromising statements such as his ‘savage review of a foreign rival’ that put sand in the gears of getting his own work published and given due credit. Nonetheless, he persisted. It was a work that, despite its apparent flaws, contributed to the development of collections research, and, like most of Sherborn’s other contributions, is still in use.

Shifting to a taxon-specific focus, Edward Dickinson presents a detailed scrutiny of Sherborn's and Richmond’s indexes in ornithology, a taxonomic best-case that illuminates problems that need attention in the larger whole of the corpus. He underscores that nomenclature is the un- (or under-) recognized foundation of taxonomy, thus calls on ornithologists to mobilize and collaborate to get the house in order for names of the approximately 10,000 bird species, as they are arguably the most public-facing of popular animal groups. This will require a level of attention to detail and collaboration that raises the game from previous ways ornithologists have worked.

F. Christian Thompson and Thomas Pape explain how research on the important (and beautiful!) megadiverse insect group Diptera has benefited from building an outstanding bibliographic index based on Sherborn’s original work. As there are 160,000 currently recognized species of flies, with over 250,000 names, this group makes up a significant proportion of planetary diversity (an estimated 10%). *Systema Dipterorum* (http://www.diptera.org/) benefits from modern tools and additions that provide a resource of greater utility than even Sherborn could have imagined.

## Current tools and innovations for bringing legacy information into the modern age

The Smithsonian Libraries have made Sherborn’s *Index Animalium* freely accessible online (http://www.sil.si.edu/digitalcollections/indexanimalium/). Suzanne Pilsk, Martin Kalfatovic and Joel Richard describe how this was done, and how the transition from paper to bytes is the dawn of a new age for bibliographic information. They point out that traditional library metadata for a book title, which was sufficient to retrieve a physical book from the stacks, is now not fit-for-purpose for the vastly increased but distributed constituencies that modern libraries serve. Rising to the challenge, the Smithsonian converted the 420,000 entries of *Index Animalium* to a detailed, fine-granularity bibliographic data set that can be used by researchers with greater speed and fidelity than print ever allowed.

A key concern for any data source is its error rate and identifying where the errors are concentrated. Francisco Welter-Schultes, Angela Görlich & Alexandra Lutze undertook a detailed study of error rates in Sherborn’s magnum opus by comparing samples with their own large team project on original sources through the project AnimalBase (http://www.animalbase.org/). They found that Sherborn’s error rate was remarkably low, at 1–2% or even less for individual entries. They point out that, ‘this is low for any human endeavor, let alone one of such monumental scale requiring detailed work over many decades. It is all the more impressive when we realize that today we have comparable failure rates, despite having computer tools and teams of people to help with this kind of work.’ However, there are areas where errors are concentrated such as names from chaotically organized original sources, from publications in languages that Sherborn did not speak, and from particular sources or taxa. Oddly, although Sherborn’s error rate for molluscan names was higher than for insect names overall, his error rate for Fabricius, the largest source of insect names, was surprisingly high. The authors advise that *Index Animalium* not be used to determine correct authorship of a name, but original sources should be consulted because they see an unacceptable level of problems with rendition of authors. Welter-Schultes, et al., suggest that a 2–4% error rate is an intrinsic limit in manually compiled data of this kind at this scale; below that there are diminishing returns. They brought this point home in a criticism of the idea of Lists of Available Names (LANs, below) and as a caution to large-scale data input into projects like ZooBank (below).

In a paper that tackles technical issues, but with a good grounding in philosophical issues, Christopher Lyal describes the limitations of digitizing objects and information. His target is bringing legacy taxonomic literature into the digital sphere. Lyal underscores our current tendency to build forward from the past, using e-charged traditional methods to produce digital analogues of paper, rather than developing new tools that make the most of cybertechnology and assessment of future needs and opportunities. He explains how text mark-up with XML can open the door to allowing not only a viewable and searchable original text, but more powerfully, for subsets to be viewed, extracted and separately analyzed. This allows repurposing in a multitude of different contexts, extending the reach of the original publication, and creating new ways of using scholarly information. It also allows automatic population of large-scale information sources such as ZooBank or GBIF. Mark-up allows dynamic linking of new and extant information. In essence, it is what Sherborn was aiming to do with *Index Animalium*.

In a paper that should become required reading for all taxonomists, David Remsen takes the issues of taxonomic knowledge bases and systems of names to their philosophical foundations. Names are a handle or tag on larger sets of concepts (taxonomy) that can be fluid, however names also link to an objective standard, a type specimen, and have a single birthplace, or point of origination, in a publication. While dealing with unstable relations between these entities is a headache, Remsen explains with crisp language and clear diagrams how it can be clarified. ‘Semiotics provides a general model for describing the relationship between taxon names and taxon concepts. It distinguishes syntactics, which governs relationships among names, from semantics, which represents the relations between those labels and the taxa to which they refer.’ He places nomenclature in the context of a graphical triangle of reference, or semiotic triangle, as a model of how syntax and semantics are related to the objects they represent. The paper provides a critical link between lists of names, like *Index Animalium* and lists of species, which are the ultimate currency that interest most users.

With a more pragmatic perspective on issues of stability of scientific names of animals, Miguel Alonso-Zarazaga, Daphne Fautin and myself detail the requirements and opportunities for Lists of Available Names (LANs) to proceed through ICZN Article 79 to stabilize large taxonomic sections of nomenclature at once. Although it is not a light task to implement a LAN, a result is that ‘nomenclatural archaeology’ will find the footing pulled out from under it, thus increasing stability and transparency in scientific names of animals. Our short, succinct paper outlines the results of deliberations of several ICZN committees. It is supported by publication of a ‘*Manual for proposing a Part of the List of Available Names in Zoology*’ by Miguel Alonso-Zarazaga, Philippe Bouchet, Richard Pyle, Nikita Kluge, Daphne Gail Fautin as an appendix to this volume. We hope these practical tools will result in well-documented, collaborative work by sectors of the taxonomic community to stabilize names that might otherwise create problems for information retrieval.

## The future of biological nomenclature, including innovative publishing models and the changing sociology of science in taxonomy

The future for taxonomy is intimately bound with the future of publishing, and biological knowledge is on the cusp of a radical change in how it is delivered and archived. The atomization and automation of publications will make step changes in how information is used. Lyubomir Penev and 11 co-authors provide a very practical glimpse of what revolutionary e-tools look like, presenting a new work flow and publishing mechanism developed by this journal, *ZooKeys*. Their paper is a collaborative approach between four lead indexes of taxon names and nomenclatural acts, and thus achieves an additional objective of harmonizing practice across taxonomic disciplines. They point out how technical tools can radically change the landscape for the persistent, and previously intractable, controversies of registration and e-publication across all biological nomenclature.

In ‘Surfacing the deep data of taxonomy’ Rod Page observes that ‘Names by themselves are of little value; it is the literature, specimens and data derived from those specimens that are the primary data of taxonomy. Yet much of this information remains hard to obtain (even discovering it exists can be challenging)’. His paper is a manifesto, targeted at a critical technical tool to achieve this goal – the form of the persistent digital link between units of information, the bibliographic identifiers. Because the revolution in digital information access has grown up through individual innovation and is facing a kind of free-market competition, not top-down infrastructural planning, we are currently in a situation where different projects have opted to use different kinds of identifiers (DOIs, or digital object identifiers, versus LSIDs, or Life Science Identifiers). Page makes a strong case that his preferred identifier system, DOIs, is the only one with the supporting features that allow complete, deep, linkage of all the primary taxonomic data. He suggests that the tracking features of DOIs allow them to potentially solve a huge problem for taxonomists in providing altmetrics that demonstrate the long and wide reach of their work. This can give taxonomists greater credit, countering the current skewed recognition based on journal impact factors. He also suggests that this bibliographic issue holds solutions to the problem of how to recognize ‘dark taxa’, those known from (usually molecular) data, but not recognized with a formal name. Page is known as a boat-rocking, take-no-prisoners provocateur; we are lucky that he has turned his sights on bibliography – follow his arguments to find where new disruptive technology will have a major constructive effect in taxonomy.

Richard Pyle’s paper, based on his wrap-up plenary talk, makes a convincing case that, even in this time of major technological improvements for all taxonomic research tools, the greatest wholesale revolutionary change is the means by which we manage and communicate information. Names are at the nexus of that revolution. Pyle puts Sherborn’s work at the center of the task of identifying and making order of our knowledge of biodiversity:

‘the Linnaean nomenclatural system [is] a stable scaffold against which the ever-changing landscape of [taxonomic] species can be referenced. … In stark contrast to the dynamic, on-going, and seemingly endless debates about what a “species” is, the nomenclatural system used by taxonomists during the past two and a half centuries has been remarkably consistent, universal, and stable….. Whereas the majority of the nearly 4,400 species circumscriptions described by Linnaeus in his 1758 *Systema Naturae* bear very little resemblance to the species boundaries asserted by modern biologists, most of the scientific names he established are not only available under the current Code, but are in current use. …Although catalogs of species (e.g., Linnaeus 1758) may begin to lose their taxonomic relevance almost immediately after publication, the scientific names established within such catalogs retain their nomenclatural relevance indefinitely. Ultimately, this is why the career-long labors of Sherborn have retained their value well beyond his own life, up until today and continuing indefinitely into the future.’

Pyle exhorts that we are now responsible for the next iteration of this Linnaean enterprise in a new way. The new paradigm for all nomenclature projects is the Global Names Architecture (GNA), the dynamic index to interconnect and streamline the entire taxonomic enterprise through names. Pyle underscores the constructive collaboration of all the major taxonomic resources (e.g., GBIF, CoL, IPNI, EoL, ZooBank) to build a dynamic suite of web services to connect them all through the GNA. Together, this is ‘the digital equivalent of the card catalogue of life – audacious task started by Linnaeus, dramatically extended by Sherborn’. Pyle convinces us that now we can now make another big leap in the content we cover, to encompass the entire living world using a consistent, interoperable information system that is accessible to all.

## Concluding remarks

We are on the brink of a new and truly open taxonomy. The revolution has arrived through the development of technical tools that open up ways to atomize information and make it quickly findable, retrievable and recombinable. New ways of working and new results will result in a taxonomic ‘Modern Synthesis’. Proactive collaboration will arise more fluidly between different systems with overlapping content. The philosophical underpinning of the mutual support between the flexibility of taxonomic interpretation and the stability of nomenclatural frameworks is becoming easier to define through appropriate bibliographic tools. Similarly, past differences in how the major taxonomic codes deal with names are being decreased through use of shared technical tools and major infrastructures collaborating for information access. Registration of new names is on an active track for implementation in several taxonomic disciplines, some with a common framework. The authors of this volume have taken different approaches to the problems for animal names faced by Sherborn, but it adds up to a multifaceted and powerful approach for all biological nomenclatural issues.

**Figure 3.**
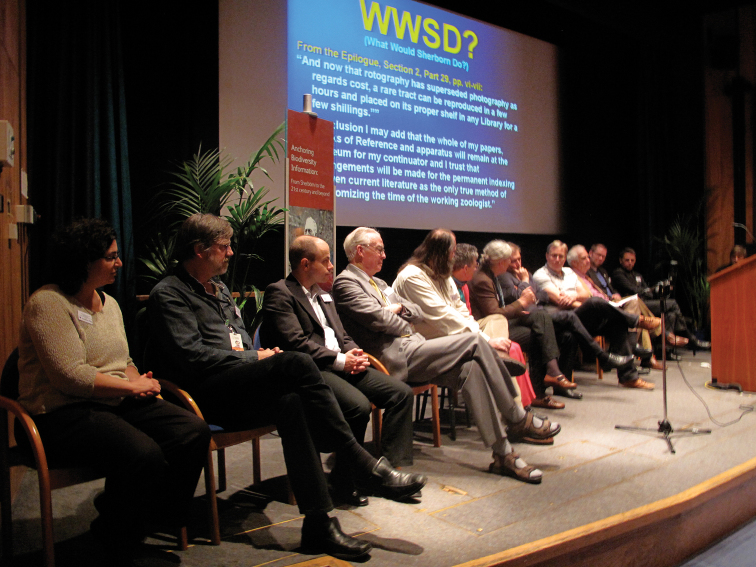
The full panel of symposium speakers under the heading **WWSD? What would Sherborn Do**? From left to right: Suzanne Pilsk, Chris Lyal, Henning Scholz, Edward Dickinson, Neil Evenhuis, Daphne Fautin, Sandy Knapp, Lyubomir Penev, Rod Page, Chris Thompson, Chris Freeland, Gordon McOuat, (behind podium Richard Pyle, David Remsen).

At the end of the symposium that gave birth to this volume, we held a panel discussion under the banner ‘WWSD? or ‘What Would Sherborn Do?’ With the contributions published here we now know much more about Sherborn as a man and scientist, about the long running nature of debates, about the current tools for making progress, and the bright future for the field. The answer is that Sherborn would have celebrated the new tools for this ambitious goal of linking all biological information through names, both machine and human readable. He would have understood its tremendous power for biodiversity science overall. And he would have knuckled down and got to work to make it happen.

